# A comparison of conventional tube and EndoFlex tube for tracheal intubation in patients with a cervical spine immobilisation

**DOI:** 10.1186/1757-7241-21-79

**Published:** 2013-11-22

**Authors:** Ewelina Gaszynska, Michal Stankiewicz-Rudnicki, Andrzej Wieczorek, Tomasz Gaszynski

**Affiliations:** 1 Department of Hygiene and Health Promotion, Medical University of Lodz, Lodz, Poland; 2 Department of Emergency Medicine and Disaster Medicine, Medical University of Lodz, Lodz, Poland; 3 Department of Anesthesiology and Intensive Therapy, Medical University of Lodz, Lodz, Poland

**Keywords:** Intubation, Cervical collar, EndoFlex

## Abstract

**Background:**

The EndoFlex is a new type of tracheal tube with an adjustable distal tip that can be bent without the use of a stylet. The aim of this study was to compare a standard endotracheal tube with the EndoFlex tracheal tube for intubation in patients with simulated cervical spine injury.

**Methods:**

A group of 60 patients without any kind of the cervical spine injury, classified as the ASA physiological scale I or II and qualified for elective surgery procedures were intubated with the use of classical Macintosh laryngoscope, and either a standard endotracheal tube with the intubation stylet in it or EndoFlex tube without stylet. The subjects were randomized into two subgroups. All patients have had the cervical collar placed on their neck for the simulation of intubation procedure in case of the spinal injury.

**Results:**

The intubation procedure was performed by 16 anesthetists with different experience (5-19 yrs). Time of intubation with the use of EndoFlex tube was similar to that with a the use of standard endotracheal tube and intubation stylet: Me (median) 19.5 s [IQR (interquatile range) 18-50] vs. Me 20 s [IQR 17-60] respectively (p = 0.9705). No significant additional maneuvers were necessary during intubation with the use of EndoFlex tube in comparison with standard endotracheal tube (70% vs. 56.6%) (p = 0.4220). Subjective assessment of the usability of both tubes revealed that more anesthesiologists found intubations with the use of EndoFlex more demanding than intubation with conventional tracheal tube and intubation stylet. The assessment of usability: very easy 3.3% vs. 20%, easy 83.4% vs. 56.7%, difficult 10% vs. 20% and very difficult 3.3% vs. 3.3% for standard endotracheal tube with stylet and EndoFlex, respectively.

**Conclusion:**

In conclusion we asses, that the EndoFlex tube does not improve intubation success rate, in fact it requires more maneuvers facilitating intubation and was found to be more difficult to use.

## Introduction

**Figure 1:**
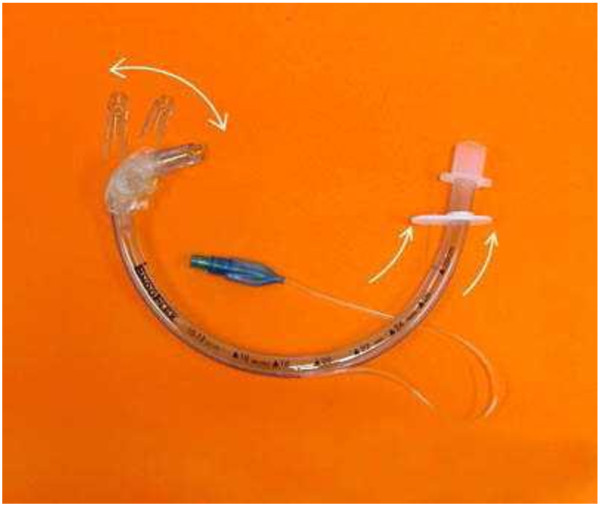
EndoFlex endotracheal tube (source: manufacturer marketing materials).

The recommended airway management strategy in trauma patients is tracheal intubation [1]. Its skilful and nontraumatic performance depends on the operator’s experience and may be exceptionally challenging in case of patients with suspected cervical spine injury. During intubation of these individuals it is crucial to minimize head movement and keep the head stabilized in-line with the trunk of the body. Intubation with the use of standard laryngoscope may not be possible without the temporary removal of cervical collar to enable head and neck movements while trying to visualize the larynx. The anatomic studies that mimic complete C4-5 ligamentous injury demonstrated that manual in-line axial stabilization reduces segmental angular rotation and distraction [2]. But it is more difficult to visualize the larynx using conventional laryngoscopy during performance of the cervical spine immobilization (even with Manual In-line Axial Stabilization MIAS) [3]. The view obtained during laryngoscopy in the presence of a cervical collar is even considerably poorer than that obtained during manual in-line immobilization. The use of a semi-rigid cervical collar has been shown to increase the incidence of Cormack-Lehane grades 3 and 4 laryngoscopic views (up to 64%) and decrease the interincisor distance when compared with conventional laryngoscopy [4]. New airway management devices should be helpful in such conditions. EndoFlex is a novel type of tracheal tube with an adjustable distal tip, that can be bent without the use of a stylet (Figure 1). Owing to its flexibility, the tube should preclude the need for the neck movement for effective intubation which is an element of great significance in case of suspected cervical spine injury. Scientific research has confirmed the advantages of EndoFlex, including lower risk of bleeding or reduction of the time to intubate in case of difficult intubation [4]. We hypothesized that the application of EndoFlex tube may improve intubation success rate in patients with the neck immobilized by semi-rigid cervical collar.

The aim of this study was to compare EndoFlex tracheal tube with the standard endotracheal tube provided with an intubation stylet for intubation in the case of simulated cervical spine injury patients immobilized with the use of a cervical collar.

## Methods

The study was conducted after obtaining an approval from the Ethics Committee of the Medical University of Lodz (RNN/719/12/KB, 16.10.2012, head: prof. Przedzisław Polakowski). It involved 16 anaesthetists from Barlicki University Hospital with different level of professional experience (5-19 yrs). All participants had standard 30 minutes training in use of EndoFlex and they had opportunity to intubate several patients with no difficulties using EndoFlex tube before the study. The intubation was performed using a Macintosh laryngoscope with either a standard tracheal tube (Sumi, Poland) with an intubation stylet or EndoFlex tube (Merlyn Associates, Tustin, CA) on patients without cervical spine injury presented for an elective surgery and classified as ASA I or II. All subjects gave informed consent to the study and had semi-rigid Patriot PA cervical collar (Medline, Poland) placed on their neck to simulate intubation in the case of spinal injury patients. Sizing and fitting of the collar was carried out according to the manufacturer’s guidelines supplied within the product package. Sizing and fitting of the collars was carried out by the same researcher.

**Figure 2:**
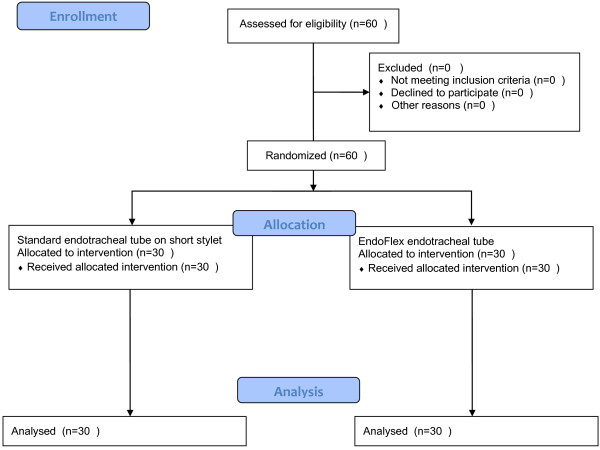
Study Flow diagram.

Using the envelope technique, 60 consecutive individuals were randomly assigned to two different groups: Macintosh laryngoscope with standard intubation stylet–STYLET (n = 30) and Macintosh laryngoscope with EndoFlex tube (n = 30) (Figure 2 Flow diagram). The sample size was not calculated but, based on literature data, we assumed that group size of 30 pts per group was sufficient for detecting differences occurring during intubation. The study was performed in a manner of a randomized, single blind, controlled clinical trial. Randomization was achieved by the following method: computer program was randomly choosing number of envelope, which was opened and the indicated method of intubation (STYLET or EndoFlex) was used in the preoperative area. Sixty envelopes were prepared, 30 for each group, and numbered after closing without knowledge of the content.

Patients were excluded from the study if the risk factors for gastric aspiration, difficult intubation (Mallampatti class III or IV; thyromental distance less than 6 cm; and inter-incisor distance less than 3.5 cm) or any other element that may deteriorate laryngeal view (for example proceeded teeth, lack of teeth, hypognatia, big tongue etc), or both were revealed. All data was collected by an independent unblinded observer.

All patients received standardized general anaesthesia: intravenous premedication consisted of 0.1 mg of fentanyl and 5 mg of midasolam. For the induction of anaesthesia, patients received 1.5 mg/kg ibw of propofol. Rocuronium (0.2 mg/kg ibw) were administrated to achieve muscle relaxance. The appropriate level of neuromuscular blockade was confirmed using 1 second stimulation (T1) with TOF-Guard device (Organon, Holland). Intubation efforts were commenced with T1 = 0. Patient’s head was always positioned in the same way for intubation on a standardized intubation pillow.

The primary endpoints were Time To Intubation (TTI) for each examined device and success rate at first attempt. Time was measured from the moment the operator took hold of the tracheal tube to the capnographic confirmation of its tracheal placement. The time elapsed was recorded by a single observer always using the same stopwatch. A failed intubation attempt was defined as esophageal intubation or situation, in which the participant is deciding to abandon the attempt. We recorded any necessity to use additional maneuvers facilitating intubation, such as external laryngeal manipulation (BURP) or different flexion of the tube’s tip. Having completed the practical part of the study, participants filled in a questionnaire concerning their subjective assessment of intubation comfort and preference for particular device.

### Statistical analysis

Statistical analysis was performed with Statistica 10.0 software (Statsoft, Tulsa, OK, USA). The Chi square test for independent pairs with Yates correction if necessary or Fisher’s exact test were used for categorical data analysis. The Shapiro-Wilk test was used to assess if variables have normal distribution and Levene test to assess the equality of variances. Mann–Whitney U test was used for non-paired categorical and continuous data analysis. P values lower than 0.05 were considered statistically significant with exception of the distribution of Cormack-Lehane comparison where Bonferroni correction for multiple comparisons lowered alpha level value to 0.0125.

## Results

**Figure 3:**
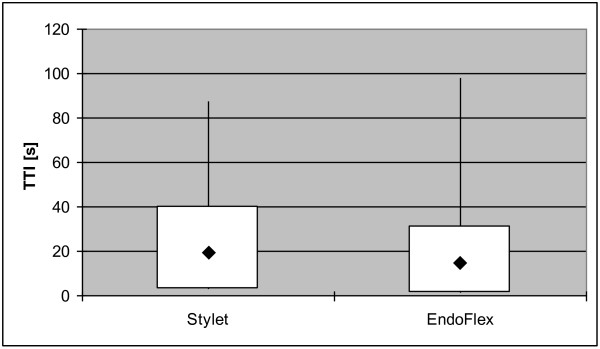
Time to intubation (TTI) with studied devices [median, IQR (box), range (lines)] (p = 0.9705).

**Figure 4:**
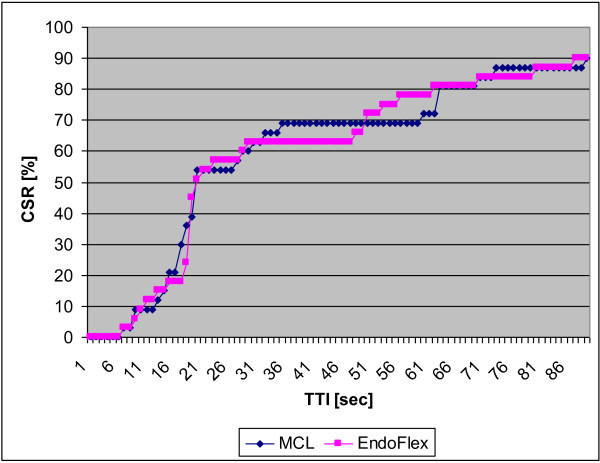
**Cumulative Success Ratio (CSR).** (TTI–Time to intubation).

**Figure 5:**
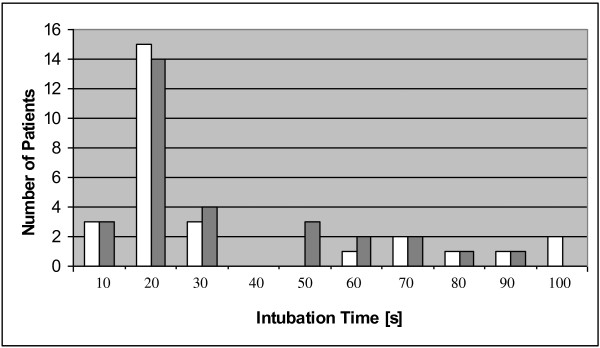
Distribution of Time To Intubation (Stylet group–white bars, EndoFlex group–grey bars).

There were no differences between groups in demographic data (Table 1). When evaluating patients airway we found that 26/30 pts in both groups had Mallampati grade 1 and no factors influencing intubation conditions. Assessment of laryngeal visualization according to Cormack-Lehane scale revealed significant differences between the two groups (Table 2). In the STYLET group, more patients had poorer visualization–CL grades III and IV. In spite of that, time to intubation with EndoFlex tube was not significantly shorter than that with a standard endotracheal tube with an intubation stylet: median 19.5 s [IQR (interquartile range) 18-50] vs. median 20 s [IQR 17-60] in EndoFlex and STYLET groups, respectively (p = 0.9705) (Figure 3). Distribution of TTI is presented in Figure 4. Twenty seven of 30 patients (90%) in each group were intubated by the first attempt. In the STYLET group, the remaining patients were intubated in the second attempt, and in EndoFlex group third attempt was needed for the remaining 3 patients. Cumulative success ratio for first attempt is presented in Figure 5. More additional maneuvers were needed during intubation with EndoFlex compared to intubation with standard endotracheal tube: 70% (21/30) vs. 56.6% (17/30) with no significant difference (p = 0.4220).

**Table 1:** Demographic data of the studied groups: mean+/-SD (standard deviation) [range]

	**STYLET group**	**Endoflex group**	**p**
Age [yrs]	49.7+/-9.6 [22-63]	46.6+/-10.3 [19-57]	0.2327
Body weight [kg]	73.4+/-10.7 [51-98]	69.58+/-10.6 [45-100]	0.1701
Height [cm]	167.6+/-8.9 [151-194]	166.16+/-8.9 [150-196]	0.5334
BMI [kg/m2]	26.2+/-3.3 [17.7-39.2]	25.2+/-5.9 [18.1-36.9]	0.4211

**Table 2:** The distribution of Cormack-Lehane score in STYLET and Endo Flex groups [% (n/n)]

	**STYLET**	**EndoFlex**	**p-value**
I	10.0% (3/30)	20.0% (6/30)	0.4716
II	50.0% (15/30)	56.7% (17/30)	0.6033
III	36.7% (11/30)	20.0% (6/30)	0.2524
IV	3.3% (1/30)	3.3% (1/30)	1.0000

P value < 0.0125 considered as statistically significant (Bonferroni correction).

Subjective assessment of the usability of both tubes revealed that more anesthesiologists found intubation with the use of EndoFlex more demanding than intubation with conventional tracheal tube and intubation stylet. The assessment of usability: very easy 3.3% vs. 20%, easy 83.4% vs. 56.7%, difficult 10% vs. 20% and very difficult 3.3% vs. 3.3% for STYLET and EndoFlex, respectively.

## Discussion

The use of EndoFlex did not shorten the time of intubation in patients with semi-rigid cervical collar neck immobilization. Cervical collar is a standard immobilization device for patients with suspected cervical spine injury–and in many cases of patients with deterioration of respiratory sufficiency, the possible necessity of collar removal before intubation may expose them to possible complications. It is recommended to use tracheal tubes with stylets when attempting intubation in individuals with suspected cervical spine injury [2]. Our study revealed that application of EndoFlex tube increases the need for additional maneuvers, such as cervical collar loosening or changes in head positioning. This is contrary to the findings of the study by Yamakage et al. [5]. He concluded that intubation with EndoFlex allowed for fewer additional maneouvres than intubation with a standard tube and enabled better laryngeal visualization. As a result, Endoflex increased chances for intubation in a relatively shorter time. However, Yamakage et al. conducted their study in patients with no cervical spine injury. Discrepancy between the two studies is most probably attributable to the fact that a cervical collar makes good laryngeal visualization more difficult.

Participants of our study found intubation with standard tracheal tube with a stylet more reliable. Analysis of the time of intubation for patients with cervical spine immobilization showed that the use of EndoFlex did not reduce it significantly despite better laryngeal visualization in the EndoFlex group, i.e. greater percentage of CL grades I and II. This may require some explanation: first of all, the difference in CL grade between groups despite no differences in airway assessment before intubation may happen because no pre-anesthesia airway test has 100% accuracy to detect possible deterioration in glottis view. In our study in both groups the same technique was employed to expose glottis to direct line of sight. Secondly, the difference in CL grade could be explained by possible influence of semi-rigid cervical collar application. The deterioration of the glottis view it causes would differ, depending on the scale used an individual; anatomy of particular patients–this may need further study. What is interesting, deterioration in CL scale in STYLET group did not prolong time of intubation compared to EndoFlex group, which may support conclusion that EndoFlex is not superior to an intubation stylet in patients with immobilized neck. Conversely, in a study of 50 patients, Teoh et al. [6] reported mean time of intubation of 30.5 s with standard tube and 14.8 s with EndoFlex, which is twice shorter than in our study. The difference is again a result of the characteristics of the examined groups. Teoh et al. examined patients with no predictible factors for difficult intubation. Our study involved patients potentially difficult to intubate because of cervical collar use.

There are only a few papers accessible on the use of EndoFlex in patients with predictable difficult intubation that confirm its efficiency. Eldawlatly reported a case of a 32-year old male with cervical osteoarthritis limiting neck movement and making standard intubation difficult. In order to intubate this patient, EndoFlex tube was combined with WuScope videolaryngoscope [7]. Similarly, Phua et al. described the use of EndoFlex tube in combination with GlideScope videolaryngoscope [8]. EndoFlex tube was declared a good alternative to standard tracheal tube and is believed to cause fewer injuries of the laryngeal inlet [9]. In our study the use of EndoFlex tube resulted in greater necessity to use additional maneuvers facilitating intubation. The number of the necessary additional maneuvers facilitating intubation was counted in system “yes” or “no”. We did not classify those maneuvers, especially that mostly all manipulations were lumped together. We assume that differences in the number of necessary additional maneuvers were related to the use of the tested methods of intubation: an intubation stylet or EndoFlex.

There are important limitations regarding our study. Firstly, we decided to impose exclusion criteria because we wanted to investigate the difficult intubation scenario (MIAS) and we did not want to potentially confound the data with other difficult scenarios. Secondly, it is impossible to blind the anaesthetist to the device being used. As the direct laryngoscopy view graduation and assessment were subjective, we decided to use the Cormack and Lehane classification which has a very important advantage: it is well understood by clinicians and widely used in clinical practice. Thirdly, in our study the number of anesthetists involved is higher than in other similar studies. On one hand it is disadvantage because each of participant intubated 3-4 times only, but on other hand in our opinion it gives more practical view to the usage of new device, because in clinical practice many clinicians with different experience will use the equipment. The judgment of one or two investigators may not reflect the actual value of a new device. Finally, this study was carried out by experienced users of each device. The results may differ in the hands of less experienced users like for example paramedics. In case of paramedics, success rate of intubation in prehospital condition is lower: 69.8% [10].

In conclusion we asses, that the EndoFlex tube did not improve intubation success rate, in fact it required more maneuvers facilitating intubation and was found to be more difficult to use.

## Competing interests

The authors declare that they have no competing interest.

## Authors’ contributions

TG projected the study design and contributed to acquisition of data, made substantial contributions to analysis and interpretation of data, drafted the manuscript, and revised it critically for important intellectual content. EG, MSR, AW contributed to acquisition of data, and revised it critically for important intellectual content. TG performed the statistical analysis and contributed to interpretation of data. MSR and AW translated the manuscript into English version. All authors have read and approved the final manuscript.
